# A new model of heart failure with preserved ejection fraction using external radiation therapy in male rats

**DOI:** 10.14814/phy2.70900

**Published:** 2026-05-12

**Authors:** Mona Guetlin, Hanan Rida, Christophe Simard, Nicolas Delcroix, Kevin Prigent, Alexandre Lebrun, Michael Joubert, Alain Manrique

**Affiliations:** ^1^ UR4650 PSIR Université Caen Normandie Caen France; ^2^ Diabetes Care Unit Caen University Hospital Caen France; ^3^ UAR3408/US50 Cyceron CNRS, INSERM, Université Caen Normandie Caen France; ^4^ Nuclear Medicine Department Caen University Hospital Caen France

**Keywords:** cardiotoxicity, heart failure, preserved ejection fraction, radiotherapy, radiation

## Abstract

Heart failure with preserved ejection fraction (HFpEF) accounts for nearly half of heart failure cases and is characterized by diastolic dysfunction, myocardial fibrosis, inflammation, and endothelial alterations. However, relevant preclinical models remain limited. As chest radiotherapy induces cardiac fibrosis and endothelial injury, we hypothesized that targeted cardiac irradiation could reproduce key features of HFpEF. Male Sprague‐Dawley rats were randomized to receive cardiac irradiation at 10 or 20 Gy, or no irradiation. Cardiac structure and function were assessed longitudina ly by echocardiography and invasive pressure‐volume Disclaimer: This is a confidential document. analysis, and exercise capacity was evaluated using a treadmil test. Myocardial fibrosis, inflammation, and oxidative stress were analyzed by histological and biochemical methods. Irradiated rats preserved systolic function but developed significant diastolic dysfunction, with increased left ventricular end‐diastolic pressure and prolonged relaxation time constant. Exercise tolerance was reduced by approximately 25% at 5 months. Histological analyses revealed increased interstitial and perivascular fibrosis with elevated co lagen I expression. CD68‐positive macrophage infiltration was markedly increased, whereas CD163‐positive ce ls were unchanged. Oxidative stress was evidenced by reduced superoxide dismutase activity and increased protein carbonylation. Targeted cardiac irradiation therefore induces a reproducible HFpEF‐like phenotype, providing a relevant model to investigate HFpEF pathophysiology and radiation‐induced cardiotoxicity.

## 1 INTRODUCTION

Heart failure (HF) is a heterogeneous clinical syndrome associating symptoms (dyspnea, excessive fatigue or muscle weakness) and clinical signs (elevated jugular venous pressure, pulmonary crackles, and peripheral edema) related to impaired cardiac performance. According to recent guidelines (TA et al., 2021), patients with HF are divided into three groups: heart failure with preserved ejection fraction (HFpEF) defined as left ventricular ejection fraction (LVEF) >50%, heart failure with reduced ejection fraction (HFrEF), defined as LVEF ≤40%, or mildly reduced ejection fraction (with LVEF between 41% and 49%). About 50% of hospitalized HF patients suffer from HFpEF (Benjamin et al., 2018), with an increasing prevalence due to population aging, with a 5‐year survival similar to HFrEF (Tsao et al., 2018).

The diagnosis of HFpEF is suggested when symptoms and signs of HF are associated with a preserved ejection fraction (≥50%) and evidence of diastolic dysfunction, which refers to impaired ventricular filling and relaxation, leading to increased pressures in the left atrium and in the lungs. In recent years, a new paradigm has emerged considering HFpEF as a multifactorial disease resulting from complex interactions between different physiological systems, including metabolic changes, endothelial dysfunction, inflammation, and fibrosis (Cornuault et al., 2022; Paulus & Zile, 2021). Especially, high levels of inflammatory cytokines such as IL‐6 and TNF‐alpha in patients with HFpEF suggested the role of inflammation in cardiac fibrosis that further contributes to ventricular stiffness and diastolic dysfunction (Paulus & Zile, 2021).

Chest radiotherapy can damage heart tissue, especially muscle and blood vessels, causing late cardiac dysfunction (Banfill et al., 2021). Late side effects depend on various factors, such as total radiation dose, fractionation, and irradiation area (Boerma et al., 2016). Structural alterations observed in the heart after chest radiotherapy may include myocardial fibrosis, altered vascular architecture, and inflammatory infiltration, which may also impair diastolic function (Boerma et al., 2016; Saiki, Petersen, et al., 2017). In addition to fibrosis, endothelial damage caused by radiotherapy can also disrupt the regulation of diastolic relaxation (Wijerathne et al., 2021). Thus, chest irradiation can affect heart function by reproducing several features of the complex pathophysiological mechanisms of HFpEF.

In clinical radiotherapy, efforts are made to minimize cardiac exposure, with recommended mean heart doses generally below 5–10 Gy and ideally <8 Gy depending on treatment protocols (Advani et al., 2026; Darby et al., 2013). Epidemiological studies have shown that the risk of cardiovascular events increases linearly with the mean heart dose, with no clearly established safe threshold (Darby et al., 2013). In experimental models, higher single doses (typically 10–20 Gy) are often used to induce reproducible cardiac injury within a shorter experimental timeframe. Direct comparison between human and animal radiation doses is challenging because of differences in cardiac size, radiation geometry, fractionation schemes, and lifespan (Asnani et al., 2021; Walls et al., 2022).

Many animal models have been proposed to mimic HFpEF, using surgical techniques, gene modifications, and pharmacological approaches (van Ham et al., 2022). However, the use of these models suffers from several limitations. First, a consistent evaluation of clinical symptoms and signs of HF is often lacking in animal models, mostly due to technical issues in evaluating exercise tolerance or fatigue. Then, ultrasound parameters widely used in humans to assess diastolic dysfunction are heavily influenced by the high heart rate in animals (300–500 bpm in rodents). Transmitral flow velocities depend on the duration of the diastolic filling phase and may vary with the type of anesthesia (Roth et al., 2002; Stein et al., 2007). Therefore, the invasive approach using pressure and volume measurements remains a gold standard to characterize diastolic dysfunction in preclinical models.

Previous experimental work by Saiki, Moulay, et al. (2017) demonstrated that a β‐targeted internal cardiac irradiation using [^131^I]‐iodine can induce a HFpEF‐like phenotype in rodents, characterized by diastolic dysfunction with preserved ejection fraction. Building on these findings, we hypothesized that a simplified methodology using external beam cardiac irradiation may yield similar results. Thus, in this study, we aimed to characterize a HFpEF model based on the use of cardiac irradiation in rats, using invasive assessment (pressure‐volume loops), treadmill exercise test, echocardiography, cardiac MR, and histology.

## 2 RESERCH DESIGN AND METHODS

### 2.1 Animals

All animal experiments and procedures were performed according to the European Commission Directive 2010/63/EU for animal care and were authorized by the French Ministry of Higher Education and Research (APAFIS#29403‐2021012916578173 v2). A total of 46 healthy males Sprague–Dawley rats (8‐week old) were purchased from Janvier Labs, and housed in cages compliant with European standards (type IV) with pathogenic‐free and controlled environments (21°C ± 1°C; humidity 60%; with a positive cage pressure 20–25 Pa relative to atmospheric pressure to maintain specific pathogen‐free conditions; lights on 6:45 am to 6:45 pm; enriched environment). All rats were fed with standard rat diet and a free access to water. Only male rats were used to avoid interference with sex hormones. They were randomized into two groups receiving (irradiated group “IR,” *n* = 30) or not a cardiac irradiation (non irradiated group “NIR,” *n* = 16). In the IR group, animals received either a radiation dose of 10 Gy in one single fraction (IR 10Gy, *n* = 15) or a dose of 20 Gy administrated as 2 fractions of 10 Gy each (IR 20 Gy, *n* = 15).

### 2.2 Anesthesia

Irradiation procedure, echocardiography, and hemodynamic measurements were performed under gas anesthesia. Anesthesia was induced using isoflurane (AbbVie, Rungis, France) at a concentration of 3% and maintained at 1.5% ± 0.5%, delivered in a gas mixture of 30% oxygen and 70% nitrous oxide (N_2_O).

### 2.3 Irradiation

Irradiation was completed using an X‐RAD 225Cx dedicated preclinical irradiator coupled with a cone‐beam CT (Precision X‐ray Inc., North Brandford, CT, USA; CYCERON biomedical imaging platform, Caen, France) in order to target the irradiation to the heart. Each irradiation session consisted of three steps: acquisition of thoracic CT simulation, dose planning, and irradiation delivery. Rats were placed in a prone position in a heated rat cradle while under gas anesthesia. The procedure began with the acquisition of a CT scout view to define irradiation ballistics. Heart‐targeted dose planning was carried out using SmartPlan® (Precision X‐ray Inc., North Branford, CT, USA), with the planning target volume (PTV) defined as the heart, without margin. Irradiation consisted of either a single 10 Gy dose or two 10 Gy fractions (20 Gy total) administered 1 week apart. To minimize exposure to the trachea and esophagus, the irradiation was delivered using two oblique beams of 5 Gy each, angled at 45° and 315°, with a 15 mm diameter collimator. Each beam lasted approximately 60–65 s, for a total session duration of 120–130 s.

### 2.4 Exercise stress test

Exercise stress test was performed at 3‐ and 5‐month follow‐up using a treadmill (LE8710, Panlab, MA) equipped with a mild electrical stimulus grid to encourage running when necessary. Each rat was running in an individual corridor, and exercise was quantified using the SeDaComv2.0.00 software (Panlab, MA). To assess exercise tolerance, only the last 3 steps were analyzed, the four first corresponding to the warm‐up. Procedure lasted about 30 min. After a 2‐min warm up at 13 cm/s, the animals were subjected to exercise at 9.6 cm/s followed by increments of 1.8 every 5 min (without slope increase) until exhaustion, which was determined by at least 4 electrical stimulations per minute. According to standard rodent treadmill protocols, the intensity of the stimulus was kept at the minimal level required to maintain running and exhaustion indicated the animal withdrawal from the test. Animals were subjected to a training session according to the same protocol 1 week before the stress test. Running distance was recorded.

### 2.5 Echocardiography

Echocardiography was performed using an iE33 ultrasound system connected with a linear high‐frequency L15‐7io ultrasound transducer (Philips Healthcare, Best, the Netherlands) at baseline (before irradiation), 3 and 5 months after irradiation. Each animal was placed on a heating plate, in dorsal recumbency, and the thorax was chemically depilated with depilatory cream (Veet®, Reckitt Benckiser, Massy, France). Ultrasound gel (AseptInmed, Quint Fonsegrives, France) was applied to the ultrasound probe. After anatomical identification in 2D mode, image acquisition was performed with the Time‐Motion (TM) mode in small‐axis view. LV end‐diastolic diameter (LVEDD), LV end‐systolic diameter (LVESD), and septal thickness at end diastole (IVSd) and end‐systole (IVSs) and posterior wall thickness at end‐diastole (PWTd) were measured by the leading edge method according to the American Society of Echocardiography guidelines. Systolic function was assessed by fractional shortening (FS = [(LVEDD − LVESD)/LVEDD]). Left‐ventricular remodeling was evaluated from LVEDD, LVESD, IVSd, and IVSs. Diastolic function was assessed indirectly by the left atrial anteroposterior diameter measured at end‐systole (LADs).

### 2.6 Cardiac hemodynamics

Under gas anesthesia, after xylocaine (5%) was sprayed over the animal neck, and buprenorphine was administrated (2 mg/kg, intramuscular), a miniaturized combined conductance micromanometer‐catheter of 2 Fr (model SPR‐838 probe; Millar Instruments) connected to a pressure‐conductance unit (MPUS‐Ultra, Millar) was advanced via the right carotid artery into the LV to measure LV end‐diastolic pressure (LVEDP), LV dP/dt, time constant of Tau relaxation. Pressure‐volume loops were recorded at baseline and during transient compression of the inferior vena cava with a cotton swab passing over the liver, allowing the calculation of the end‐diastolic pressure‐volume relationship (EDPVR) as an indicator of load‐independent LV compliance, expressed as mmHg/RVU (relative volume units). At the end of the hemodynamic measurements, animals were euthanized under deep anesthesia induced by isoflurane overdose (≥5%), followed by a lethal intra‐peritoneal injection of pentobarbital. Death was confirmed by cessation of cardiac activity. The hearts were harvested and frozen at −80° for subsequent tissue analysis.

### 2.7 Histology and immunohistochemistry

For each heart, successive 6 μm‐thick slices were collected in the long axis of the left ventricle. Slices were placed on glass microscope slides for staining. Two slices were stained with Picrosirius Red to reveal collagen (Sirius Red, RAL, #363440‐0005, Labelians, Nemours, France; Picric acid, VWR international, Rosny sous Bois, France) and Masson Trichrome (hematoxylin, Labelians; fuschin acid, RAL Diagnostics; ponceau xylidin dye, RAL, #316150‐0025; phosphomolybdic acid, VWR, #20616.184, Honeywell Fluka, Illkirch, France; light green, RAL, #320770‐0025). For immunohistochemistry, 6‐μm cryosections were stained according to standard protocols. CD68 rabbit monoclonal antibody (1:600, #97778, Cell Signaling Technology, Danvers, MA, USA) and CD163 rabbit monoclonal antibody (1:200, #93498, Cell Signaling Technology) were employed to identify macrophages. COL1A1 rabbit monoclonal antibody (1:100, #72026, Cell Signaling Technology) was utilized to assess type 1 collagen distribution. Rabbit polyclonal recombinant anti‐endothelin A receptor/ET‐A antibody (1:100, AER‐001‐200UL, Thermo Fisher Scientific, Waltham, MA, USA) and rabbit polyclonal recombinant anti‐endothelin B receptor/ET‐B antibody (1:100, #PA3‐066, Thermo Fisher Scientific) were used to identify vessels. Additionally, rabbit monoclonal vimentin antibody (1:100, #5741, Cell Signaling Technology), and rabbit monoclonal alpha smooth muscle actin antibody (1:400, #19245, Cell Signaling Technology) were employed to identify fibroblasts and myofibroblasts. Furthermore, mouse monoclonal desmin antibody (1:1200, #ab8470, Abcam, Cambridge, UK) was used specifically to identify cardiomyocytes. Secondary reagents included OmniMap anti‐Ms HRP, Roche Diagnostics for desmin and OmniMap anti‐Rb HRP (Roche Diagnostics, Basel, Switzerland) for other antibodies. Then, the ChromoMap DAB (Roche Diagnostics; ref. 05266645001) was used. After staining, heart slices were imaged using a digital slide scanner (Olympus VS120, Olympus, Rungis, France) at 20× magnification using VS‐ASW software (Olympus) and analyzed using QuPath software version 0.3.2 (University of Edinburgh, Edinburgh, UK).

### 2.8 Oxidative stress

When X‐rays interact with biological tissues, they can generate free radicals and induce oxidative stress. Oxidative stress was assessed by measuring superoxide dismutase (SOD) activity, glutathione peroxidase (GPx) activity, vitamin E levels, lipid peroxides, and protein carbonyl levels in plasma using an automated clinical chemistry analyzer (RX Imola, Randox Laboratories, Crumlin, UK). SOD activity was determined using a colorimetric assay based on the inhibition of superoxide‐driven reactions according to the manufacturer's protocol. Protein carbonyl levels were measured using a colorimetric assay based on the reaction with 2,4‐dinitrophenylhydrazine (DNPH), according to the manufacturer's instructions. Results were expressed in U/L (SOD) or nmol/mg protein (protein carbonyls).

### 2.9 Statistical analysis

Data are expressed as mean ± SEM. The distribution of variables was tested using a normality test (Shapiro–Wilk test). Parametric (paired or unpaired *t*‐test) and non‐parametric tests (Wilcoxon or Mann and Whitney) were used when appropriate. Quantitative parameters' evolution over time was analyzed by a linear time model. Qualitative variables were compared by *χ* ^2^ test. All analyses were performed using JMP 11 (SAS institute, Cary, NC). A *p*‐value ≤0.05 was considered statistically significant.

## 3 RESULTS

Two irradiation regimens were tested (10 Gy single dose and 20 Gy in two fractions). Subgroup analyses did not reveal significant differences between these two groups for the main functional, structural, and histological endpoints. Therefore, results are presented pooled as a single IR group.

### 3.1 Exercise tolerance

**FIGURE 1:**
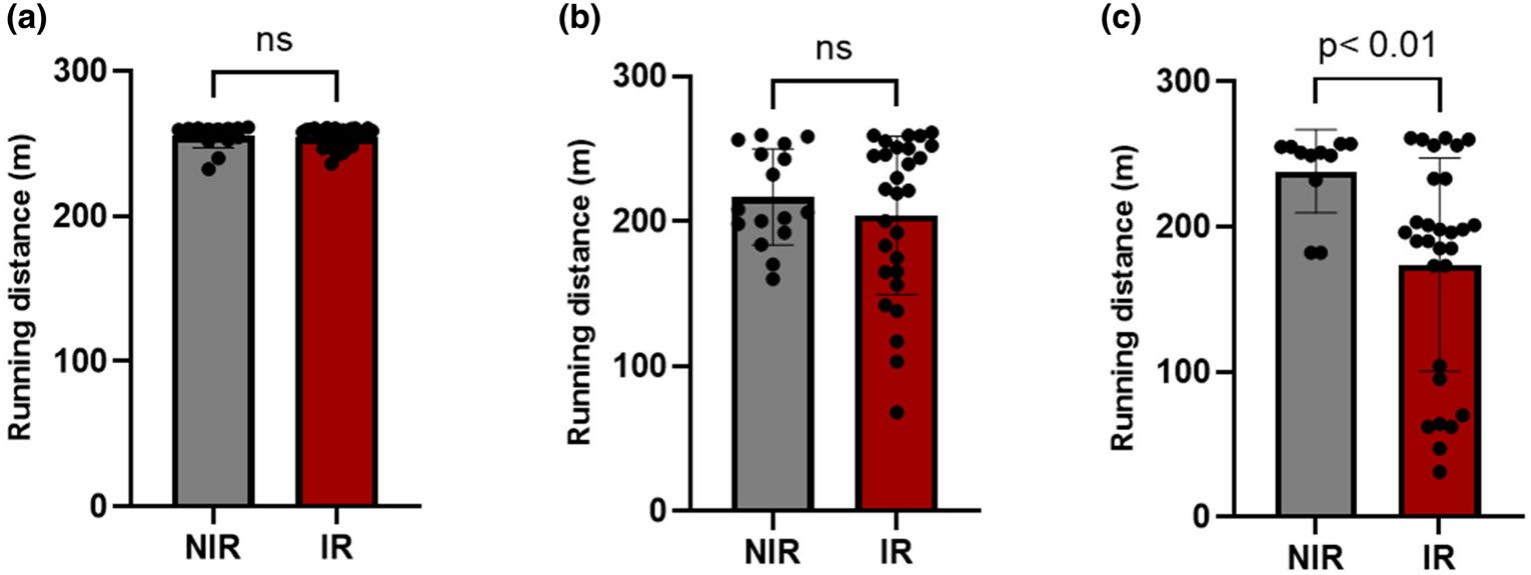
Running distance during treadmill running in non‐irradiated (NIR) and irradiated (IR) rats at baseline (a), 3 months (b), and 5 months (c) follow‐up. Data are expressed as mean ± SEM.

At baseline, the running distance was similar between non‐irradiated (255.6 ± 2.1 m) and irradiated animals (254.1 ± 1.3 m, *p* = ns). Three months after irradiation, the running distance was similar between irradiated (202 ± 11 m) and non‐irradiated animals (211 ± 10 m, *p* = ns). At 5‐month follow‐up, irradiated animals exhibited a decreased running distance indicating an impaired exercise tolerance, whereas it remained unchanged in the non‐irradiated group (217 ± 18 m vs. 149 ± 19 m, *p* < 0.01) (see Figure 1).

### 3.2 ECG

The comparison of surface ECGs between the two groups did not show any differences: in particular, there were no signs of left ventricular hypertrophy (LVH), no repolarization abnormalities, and no conduction disturbances (see Table 2). Compared to NIR animals, IR rats exhibited a decreased SDRR (3.27 ± 0.37 ms) compared to IR (5.6 ± 0.9 ms, *p* = 0.03) and an increased LF/HF ratio (0.7 ± 0.2) compared to IR rats (0.21 ± 0.05, *p* = 0.05), both indicating an increased sympathetic tone.

### 3.3 Left ventricular systolic function

Echocardiographic assessment of left ventricular systolic function was similar between IR and NIR animals at both baseline and 5‐month follow‐up. An increase in LVEDD, LVESD, and septum was noted over time in both groups corresponding to the animal growth (see Table 1). In addition, complementary invasive pressure‐volume analysis found no significant difference in dP/dt_max_ between IR (6139 ± 706 mmHg/s) and NIR (5852 ± 879 mmHg/s, *p* = ns) groups.

**TABLE 1:** Echocardiographic assessment of left ventricular remodeling and systolic function at baseline, 3 months, and 5 months in non‐irradiated (NIR) and irradiated (IR) rats.

	Baseline		3 months		5 months		*p*‐value		
	NIR *n* = 16	IR *n* = 30	NIR *n* = 16	IR *n* = 28	NIR *n* = 11	IR *n* = 19	Global	Time	Irradiation
LVEDD (mm)	7.01 ± 0.54	7.13 ± 0.44	8.45 ± 0.6	8.07 ± 0.59	8.29 ± 0.59	7.99 ± 0.7	<0.0001	<0.0001	<0.10 (ns)
LVESD (mm)	3.52 ± 0.86	3.86 ± 0.52	3.52 ± 0.86	4.89 ± 0.8	5.26 ± 0.97	5.15 ± 1	<0.0001	<0.0001	0.93 (ns)
IVSd (mm)	2.19 ± 0.34	2.15 ± 0.4	2.22 ± 0.27	2.35 ± 0.33	2.48 ± 0.53	2.33 ± 0,57	0.18 (ns)	‐	‐
PWTd (mm)	2.93 ± 0.57	2.85 ± 0.68	2.71 ± 0.58	2.72 ± 0.69	2.93 ± 0.73	2.81 ± 0.58	:	‐	‐
LADs (mm)	4.98 ± 0.51	5.29 ± 0.8	6.35 ± 0.86	6.02 ± 1.04	5.65 ± 0.29	5.48 ± 0.39	<0.0001	<0.0001	0.74 (ns)
FS (%)	50.17 ± 9.39	45.94 ± 5.57	38.92 ± 6.71	39.6 ± 7.15	36.74 ± 9. 74	35.92 ± 8.49	<0.0001	<0.0001	0.32 (ns)

*Note*: Values are expressed as mean ± SD. *p* values are provided for the linear model (global) and each effect (time or irradiation) when appropriate.

Abbreviations: FR, fractional shortening; IVSd, interventricular septal thickness at end‐diastole; LADs, left atrial anteroposterior diameter at end‐systole; LVEDD, left ventricular end‐diastolic diameter; LVESD, left ventricular end‐systolic diameter; PWTd, posterior wall thickness at end‐diastole.

**TABLE 2:** Heart rate variability indices in irradiated (IR) and non irradiated (NIR) rats.

	NIR (*n* = 9)	IR (*n* = 18)	*p*‐value
SDRR (ms)	5.5 ± 2.85	3.3 ± 1.5	0.03
LF/HF (m^2^)	0.21 ± 0.16	0.73 ± 0.99	0.05

*Note*: Data are expressed as mean values ± SD; *p* < 0.05.

Abbreviations: LF/HF, low frequency/high frequency ratio; SDRR, standard deviation of RR intervals.

### 3.4 Left ventricular diastolic dysfunction

**FIGURE 2:**
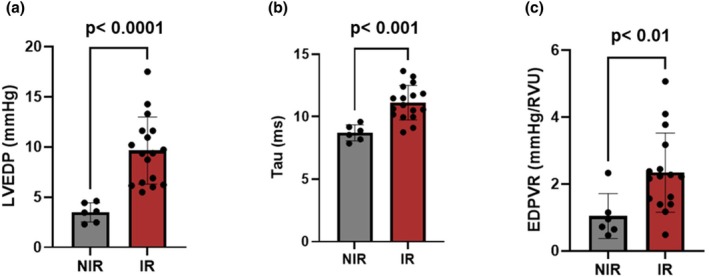
(a) Left ventricular end‐diastolic pressure (LVEDP) in non‐irradiated rats (NIR) and irradiated rats (IR). (b) Isovolumetric relaxation constant (Tau) in NIR and IR. (c) End‐diastolic pressure–volume relationship (EDPVR) in NIR and IR. Data are presented as mean ± SEM.

Echocardiography showed no significant change in left ventricular wall thickness (see Table 1). However, invasive hemodynamic measurements demonstrated a marked impairment of diastolic function in IR animals, affecting both relaxation and compliance (see Figure 2). In the IR group, there was an increased relaxation time demonstrated by a lengthening in Tau constant in IR (11.1 ± 1.4 ms) compared to the NIR group (8.7 ± 0.6 ms, *p* = 0.0004). Similarly, mean LVEDP was increased (9.6 ± 3.3 mmHg) compared to the NIR group (3.5 ± 0.9 mmHg, *p* < 0.0001). Finally, the end‐diastolic pressure–volume relationship (EDPVR) was significantly increased in the IR group (2.4 ± 1.9 mmHg/RVU) compared to NIR animals (1 ± 0.7 mmHg/RVU, *p* = 0.209).

### 3.5 Immunostaining

**FIGURE 3:**
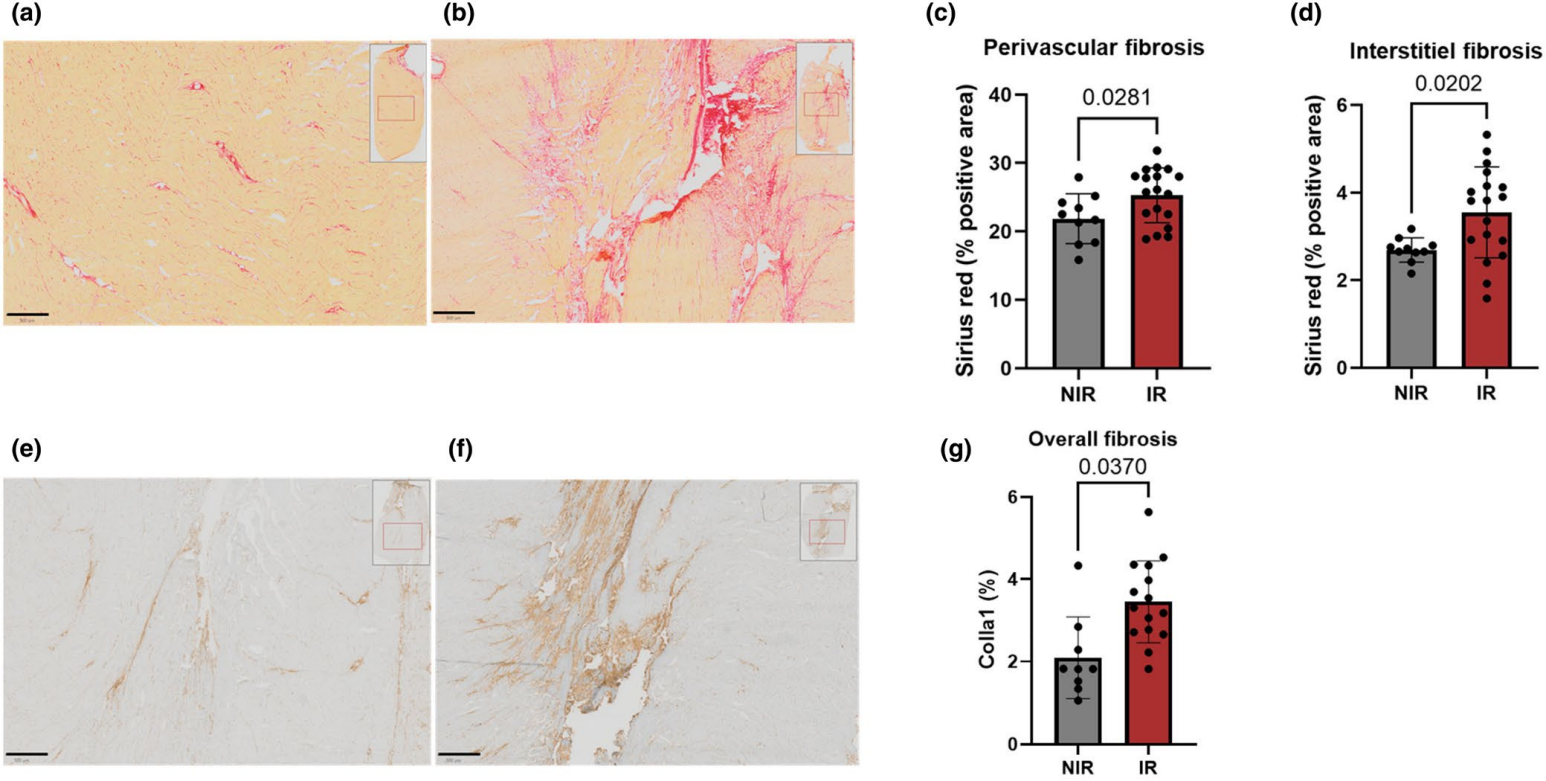
(a, b) Representative Sirius Red staining in left ventricular sections from NIR (a) and IR (b) rats, showing interstitial and perivascular collagen deposition (red). (c, d) Quantification of perivascular (c) and interstitial (d) fibrosis based on Sirius Red–positive area. (e, f) Immunohistochemical staining for collagen type I alpha 1 (Col1a1) in NIR (e) and IR (f) rats using a rabbit monoclonal antibody. (g) Quantification of Col1a1 staining confirmed increased collagen deposition in IR rats. Data are presented as mean ± SEM; The scale bar represents 500 μm.

The percentage of myocardial fibrosis detected by Sirius Red staining was significantly higher in the IR group (3.5 ± 0.2%) compared to the NIR group (2.7% ± 0.1%, *p* = 0.0202). Similarly, perivascular fibrosis was significantly increased in the IR group (25% ± 4%) compared to the NIR group (22 ± 4%, *p* = 0.0281) (See Figure 3). Collagen type I alpha 1 expression was significantly higher in the hearts of IR rats compared to NIR rats (3.435% ± 0.99% vs. 2.095% ± 0.98%, *p* = 0.037). Myocardial macrophage infiltration was significantly increased in the IR group, as shown by CD68 immunostaining (0.074% ± 0.073% vs. 0.014% ± 0.018%, *p* = 0.01), indicating a fivefold relative increase in pan‐macrophage marker. In contrast, CD163 staining, a marker of M2 anti‐inflammatory macrophages, remained low and did not differ between groups (0.021% ± 0.017% in IR vs. 0.020% ± 0.019% in NIR, *p* = 0.733).

### 3.6 Oxidative stress

In irradiated animals, superoxide dismutase activity was significantly decreased (*p* = 0.05). Then, the proteins that underwent oxidation, also called carbonylated proteins, were significantly increased in the irradiation group, suggesting an increase in free radical production induced by irradiation (*p* = 0.021).

## 4 DISCUSSION

We developed and characterized a novel preclinical model of HFpEF based on targeted cardiac irradiation. Using this approach, we showed that radiation exposure induces a HFpEF‐like phenotype with impaired diastolic function, characterized by both increased chamber stiffness (steeper EDPVR) and impaired relaxation (prolonged Tau), associated with elevated filling pressures and reduced exercise tolerance, in the absence of systolic dysfunction as evidenced by preserved EF, dP/dtmax, and fractional shortening. The diastolic abnormalities were associated with increased myocardial and perivascular fibrosis, as well as enhanced collagen type I deposition. Myocardial inflammation was also present, with a significant increase in CD68^+^ macrophage infiltration, while anti‐inflammatory CD163^+^ macrophages remained unchanged, suggesting a predominance of pro‐inflammatory cell recruitment. In addition, oxidative stress was aggravated, with reduced antioxidant activity (SOD) and accumulation of oxidized proteins. Finally, autonomic imbalance was evidenced by reduced heart rate variability and increased LF/HF ratio, consistent with sympathetic activation. Taken together, these data support the role of inflammation, oxidative stress, fibrosis, and autonomic dysregulation as converging mechanisms underlying radiation‐induced diastolic dysfunction. This innovative model provides translational insights into the pathways linking radiotherapy‐induced cardiotoxicity and the pathophysiology of HFpEF.

Our results confirm previous findings demonstrating diastolic dysfunction after β^−^irradiation. Using a complex technique associating ^131^I administration after sodium/iodine symporter transfection to deliver similar irradiation doses to the heart, Saiki et al. showed a resulting impaired exercise capacity and LV diastolic dysfunction with preserved systolic function (Saiki, Moulay, et al., 2017). Using a simplified methodology based on an X‐ray external beam which more closely mimics clinical radiation exposure, we confirm in the present study that cardiac irradiation resulted in a significant diastolic dysfunction characterized by an increase in LVEDP, Tau and EDPVR, and was associated with a preserved systolic function.

In humans, it is well known that radiotherapy for thoracic cancer such as breast, lung, esophageal cancers, and lymphomas can cause cardiotoxicity leading to both acute and chronic cardiovascular conditions, despite the use of modern radiotherapy techniques (Bergom et al., 2021; Díaz‐Gavela et al., 2021). Notably, heart failure with preserved ejection fraction (HFpEF) is increasingly recognized as a late manifestation of radiation‐induced cardiac toxicity, likely associated with diffuse myocardial fibrosis and impaired ventricular compliance (Saiki, Petersen, et al., 2017).

Cardiac irradiation induces a cardiotoxicity characterized by cellular and molecular changes that lead to diffuse interstitial fibrosis, which occurs even after relatively low doses (Roth et al., 2002; Stein et al., 2007) as low as 1 Gy (Lara, 1996). Clinical studies in breast cancer patients have shown that the risk of developing heart disease and major adverse cardiovascular events increases by 4%–16% for each Gray (Gy) of mean heart radiation dose (MHD) delivered during treatment (Darby et al., 2013; Taylor et al., 2017; van den Bogaard et al., 2017). In our study, we found an increase in myocardial and perivascular fibrosis, using irradiation doses of 10 Gy in a single fraction or 20 Gy in two fractions. Seeman et al. (Seemann et al., 2012) demonstrated that a low dose radiation delivered to the heart (2 or 8 Gy) did not impair LV systolic function. However, increasing the irradiation to a single dose of 16 Gy resulted in a significant increase in mortality. Saiki, Moulay, et al. (2017) showed in a rat model that cardiac irradiation at 10 or 20 Gy led to increased fibrosis, resulting in diastolic dysfunction characterized by elevated filling pressures measured by cardiac catheterization. Consistently with these previous findings, our analysis did not reveal major differences between 10 and 20 Gy regimens, suggesting that the pathological changes leading to fibrosis and diastolic dysfunction may already be triggered at the lower dose tested, with no further aggravation when doubling the irradiation.

Myocardial infiltration was significantly increased in the IR group, as indicated by enhanced CD68 staining, while CD163 levels remained unchanged, suggesting a predominant recruitment of pro‐inflammatory, M1‐like macrophages as opposed to CD163‐positive M2 macrophages. M2 macrophages are typically involved in tissue repair and inflammation resolution. This pattern is consistent with previous reports highlighting the central role of M1 macrophages and their activation as a key event in myocardial remodeling in patients with HFpEF (Gorica et al., 2023).

Advances in cardiovascular research have highlighted the crucial role of oxidative stress in the pathogenesis of heart failure, both in its development and progression (van der Pol et al., 2019). We have demonstrated a significant decrease in SOD activities in our model, which is consistent with several observed studies (Khaper et al., 2003; Khaper & Singal, 1997). Gomes et al. (2020) showed in a rat model that myocardial infarction increases oxidative stress and decreases SOD activity. This decrease could be explained by the existence of a close correlation between reduced antioxidant reserve and impaired cardiac function (Hill & Singal, 1996). Subsequently, we observed a significant increase in carbonylated proteins in the irradiated group compared to the non‐irradiated group. Carbonylated compounds are widely used markers of severe protein oxidation (Karimi Galougahi et al., 2015).

Finally, the measurement of sinusoidal variability, reflected by the RR interval on an ECG, showed a more pronounced sympathetic activation in the irradiated group. This is consistent with the literature: Wu et al. also demonstrated that it can induce cardiovascular autonomic dysfunction by reducing parasympathetic activity and increasing sympathetic activity (Wu et al., 2023). The suspected mechanisms would be related to direct toxicity of radiotherapy on neurons via radiation and indirect toxicity through the induced inflammation (Baselet et al., 2019). Autonomic nervous system abnormalities in heart failure are well known, and the prognostic importance of sinusoidal variability (or HRV) in cardiovascular diseases has been widely reported. Indeed, its alteration in heart failure is associated with an increased mortality rate (Lahiri et al., 2008).

A limitation, however, relates to the evaluation of exercise capacity. Exercise intolerance is a main symptom in heart failure (van Ham et al., 2022), and can be evaluated in rodents using different protocols. At best, exercise performance is evaluated on a treadmill by measuring VO2 max, which remains a complex procedure in animal models. We used a low intensity aerobic exercise protocol, adapted from published guidelines for exercise and training protocols in animals (Poole et al., 2020) and found a 25% reduction in running distance 5 months after irradiation, which is similar to previous findings in animals with chronic aortic banding (Westermann et al., 2008) or myocardial infarction (Gomes et al., 2020) compared to control.

## 5 CONCLUSION

In conclusion, our results suggest that irradiation of the heart in rats induces cardiac features consistent with heart failure with preserved ejection fraction (HFpEF), characterized by diastolic dysfunction and increased left ventricular filling pressures, likely mediated through pro‐inflammatory and pro‐fibrotic mechanisms. This experimental model may provide useful insights into the pathophysiology and progression of HFpEF.

## AUTHOR CONTRIBUTIONS

**Mona Guetlin:** Formal analysis; funding acquisition; investigation; methodology; project administration; supervision; validation; visualization. **Hanan Rida:** Conceptualization; data curation; formal analysis; methodology; validation. **Christophe Simard:** Conceptualization; data curation; methodology; project administration; resources; supervision. **Nicolas Delcroix:** Conceptualization; data curation; formal analysis; funding acquisition. **Kevin Prigent:** Conceptualization; data curation; formal analysis; funding acquisition; investigation; methodology; project administration; resources; visualization. **Alexandre Lebrun:** Funding acquisition; methodology; project administration; resources. **Michael Joubert:** Conceptualization; data curation; formal analysis; methodology. **Alain Manrique:** Conceptualization; data curation; formal analysis; funding acquisition; investigation; methodology; visualization.

## FUNDING INFORMATION

This research was partially supported by a grant from the GCS G4 in the framework of the FHU‐CARNAVAL project under the AVIESAN label.

## CONFLICT OF INTEREST STATEMENT

The authors declare no conflicts of interest.

## ETHICS STATEMENT

All animal experiments were conducted in accordance with the European Directive 2010/63/EU for the protection of animals used for scientific purposes and were approved by the French Ministry of Higher Education and Research (APAFIS#29403‐2021012916578173 v2).

## Data Availability

The data that support the findings of this study are available from the corresponding author upon reasonable request.
